# 

**Published:** 1849-05

**Authors:** 


*New Books.*—Several new publications have been received, but too late for notice in this number. We will endeavor to do them justice in our next.

*Conclusion of Volume.*—With this number the *fourth* volume of this Journal terminates. The same arrangements as heretofore will continue. The publishers desire to say that the appearance of the work will be improved by a better quality of paper than has heretofore been used, and that this number, in this respect, is a specimen of the *fifth* volume. We tender our thanks for the increased, and increasing patronage extended to the Journal, and to our correspondents for their valuable aid. The index and title page to the volume just concluded, will accompany our next number. Subscribers will please take notice of this, and not have the volume bound prior to the reception of the June number.

---

*Editorial Changes.*—Some further changes in the Editorial *corps* have recently taken place. Drs. Drake and Colescott have retired from the *Western Journal of Medicine and Surgery,* and Dr. Lunsford P. Yandell remains as the Editor, assisted by Dr. Theodore S. Bell, who was formerly connected with the work.

Dr. A. Hester is now the sole Editor of the New Orleans Medical and Surgical Journal. Dr. Harrison, formerly one of the Editors of this work, resigned on account of ill health, and is since deceased. Dr. Harrison was a talented writer, and in his death the Profession of New Orleans has sustained a loss which will be deeply felt.

---

*American Medical Association.*—Before this number is received by our readers, the meeting of the Association will have taken place. We learn from the Boston Medical and Surgical Journal, that Dr. Bowdwitch, of the committee on reception of delegates, has been notified of the appointment of a large number coming from every quarter of the Union. The attendance will probably be larger than at any previous year of the existence of the Association. We hope to be present, and shall endeavor to give some account of the proceedings in our next number.

*To Correspondents.*—Dr. Rogers' communication was received after our original department was made up for this number. It will receive insertion in the June number.

The translated report by Dr. Bryan has been received, and will be inserted in our next number. We beg Dr. Bryan to accept our thanks for his valuable contributions.

